# Effect of Viscosity on Bouncing Dynamics of Elliptical Footprint Drops on Non-Wettable Ridged Surfaces

**DOI:** 10.3390/polym13244296

**Published:** 2021-12-08

**Authors:** Sungchan Yun

**Affiliations:** Department of Mechanical Engineering, Korea National University of Transportation, Chungju 27469, Korea; syun@ut.ac.kr

**Keywords:** non-wettable surface, ridged surface, interfacial dynamics

## Abstract

An initial drop shape can alter the bouncing dynamics and significantly decrease the residence time on superhydrophobic surfaces. Elliptical footprint drops show asymmetric dynamics owing to a pronounced flow driven by the initial drop shape. However, the fundamental understanding of the effect of viscosity on the asymmetric dynamics has yet to be investigated, although viscous liquid drop impact on textured surfaces is of scientific and industrial importance. Here, the current study focuses on the impact of elliptical footprint drops with various liquid properties (density, surface tension, and viscosity), drop sizes, and impact velocities to study the bouncing dynamics and residence time on non-wettable ridged surfaces numerically by using a volume-of-fluid method. The underlying mechanism behind the variation in residence time is interpreted by analyzing the shape evolution, and the results are discussed in terms of the spreading, retraction, and bouncing. This study provides an insight on possible outcomes of viscous drops impinging on non-wettable surfaces and will help to design the desired spraying devices and macro-textured surfaces under different impact conditions, such as icephobic surfaces for freezing rain or viscous liquids.

## 1. Introduction

Drop impacting onto solid surfaces is a natural phenomenon [1,2] and essential for many engineering applications, such as spraying cooling [3], forensic application [4], pesticide deposition [5], inkjet printing [6], and impact erosion [7]. Previous studies on the liquid drops impacting on solid surfaces with various wettabilities assisted us to design surfaces for self-cleaning [8,9], anti-icing [10], and increasing the efficiency of heat exchange [11] in solar photovoltaics, condensers, and steam turbines, etc. For the past two decades, fluid repellency from the hydrophobic and superhydrophobic surfaces (SHSs) has been an active field for understating the fundamentals and developing diverse applications [8,12,13]. SHSs show outstanding anti-wetting properties characterized by the higher water contact angles (>150°) and very small contact angle hysteresis (<5°). The dynamics of the solid−liquid interactions can yield the theoretical Rayleigh limit given by 2.22 (*ρD*^3^/8*σ*)^1/2^, independent of the impact velocity, where *ρ*, *D*, and *σ* are the density of liquid, equilibrium diameter, and interfacial tension, respectively [12,13]. The theoretical limit has been accepted as the shortest residence time of symmetrical drop bouncing from SHSs [12]. The impact dynamics can be determined by the following dimensionless numbers [1,2]: Weber number, *We* = *ρDU*^2^/*σ*, Reynolds number, *Re* = *ρDU*/*μ*, Ohnesorge number, *Oh* = *μ*/(*ρDσ*)^1/2^, and capillary number, *Ca* = *μU*/*σ*, where *U* is the impact velocity and *μ* is the viscosity of liquid. The group of dimensionless numbers allows us to comprehend the relative magnitudes of the inertial and viscous forces and the surface tension. In addition, a dimensionless number, called Impact number (*P* = *We*/*Re*^4/5^), was used for a comprehensive estimation on whether capillary or viscosity governed the drop dynamics [13]. If Impact number was greater than unity, the viscosity effect was dominant in the hydrodynamics.

Water is a fluid of which viscosity can be ignored (*μ*~1 mPa s), so it has been widely used in the field of drop impact and wetting. However, effects of viscosity can become visible and significantly change the residence time at low temperatures because the viscosity of water drop increases. Unfortunately, not much attention has been paid to viscous impacts, and most of the studies have been devoted to inviscid impacts, except for the following. Mao et al. [14] studied viscous liquid drop (*μ*~1–100 mPa s) impact on surfaces with various wettabilities. They predicted the maximum spreading diameter and tendency of drop rebounding as a function of viscosity and static contact angle. Bartolo et al. [15] investigated the retraction behavior of viscous liquid drop (*μ*~1–205 mPa s) and presented inertial-capillary and viscous-capillary regimes based on Ohnesorge number. Lin et al. [16] conducted the systematical investigation on viscous liquid drop (*μ*~1–398 mPa s) impact on solid surfaces with various wettabilities, from hydrophilic to SHSs. They focused on studying maximum spreading diameter and spreading time by introducing a modified inertial-capillary time scale and Weber number. Yeong et al. [17] demonstrated viscous liquid drop (*μ*~1–8 mPa s) impact on inclined SHSs and found that, as the viscosity of the fluid increased, the receding angle of the surfaces reduced significantly, thereby altering a drop’s rebound characteristics. Abolghasemibizaki et al. [18] investigated liquid drops with various viscosities (*μ*~8–100 mPa s) and impact velocities. They reported that the drop dynamics was related to residence time on non-wettable flat and textured surfaces, and the retraction velocity could be scaled as both inertial-capillary velocity (~(*σ*/*ρD*)^1/2^) and viscous-capillary velocity (~*σ*/*μ*). Raiyan et al. [19] studied the effect of viscosity (*μ*~1–23 mPa s) on bouncing dynamics with and without a macro-ridge by investigating the conditions for observing drop splitting with various viscosities.

Recent studies reported that the residence time can be altered below the inertial-capillary time scale (*τ*_0_ = (*ρD*^3^/8*σ*)^1/2^) by macroscopic surface structures [20,21,22] and modifying the initial drop shapes [23,24,25] to challenge the limit of the time scale in symmetric bouncing. Bird et al. [20] demonstrated the residence time reduction by using a single macroscopic ridge, which induced a higher retraction velocity on the ridge and the subsequent redistribution of the drop into butterfly shapes. Afterward, Gauthier et al. [21] introduced repellent wires to SHSs to investigate the drop dynamics for several drop diameters, wire sizes, and impact velocities. They found a steplike decrease in residence time at intermediate- and high-impact velocities, suggesting the residence time relation, *t*_0_/*n*^1/2^, where *t*_0_ is the residence time of the drop on a surface without the macro-texture, and *n* is the number of lobes of drops. Patterson et al.’s [22] experimental results showed that the number of intersecting spokes had an effect on Leidenfrost drops’ residence time, and the previous relation of *t*_0_/*n*^1/2^ did not follow the experimental results when the drop was split to *n* > 2.

The control of drop mobility might be effective in the situation where the target solid must be adjustable in such aforementioned studies. However, if the target solid is uncontrollable, the target liquid or initial shape of the drop might be one of the candidates to modify the impact dynamics. The author’s previous study confirmed that the initial drop shape can alter the bouncing behavior and significantly decrease the residence time if ellipsoidal drops collided on flat surfaces [23]. Recently, the author’s previous studies proposed the asymmetry of bouncing behavior of spheroidal and ellipsoidal water drops on SHSs to demonstrate a collaboration between the initial drop shapes and ridged surfaces [24,25]. The studies reported the feasibility of shortening the residence time by ~50% using a volume-of-fluid (VOF) method, compared with symmetric bouncing of spherical drops on flat surfaces. For different geometric relationships between the drops and ridge, the non-spherical shapes induced different dynamics in the directions parallel and perpendicular to the macro-texture during the whole of the impact. However, previous explanations for residence time reduction are valid only for water drops and do not hold for viscous liquids. Furthermore, it is necessary to understand how shape distortions of viscous liquid drops affect the liquid repellency from surfaces using macro-texture in practical terms.

In this study, it is hypothesized that viscosity and initial drop shape might alter the bouncing behavior considerably and play an important role in designing the desired impinging system and surface modification for many practical applications. The current work focuses on studying the impact of ellipsoidal drops with various liquid properties (*μ*~1–100 mPa s) and drop sizes by predicting the bouncing behavior and residence time (*t_c_*) on ridged surfaces numerically, using the VOF method [26]. A rectangular shape of the ridge is chosen as a representative of a ridge because it is simple to be manufactured and widely used. The impact velocity is a very essential factor, responsible for viscous liquid’s repellency from surfaces. The numerical simulation provides a proof-of-concept for a reduction in the residence time compared with symmetric bouncing of spherical drops. The underlying mechanism behind the variation in *t_c_* is investigated by analyzing the drop dynamics. In addition, the results are discussed in terms of the spreading, retraction, and bouncing.

## 2. Materials and Methods

The VOF method was employed to predict the bouncing behavior of the drops on ridged surfaces and observe how the drops’ ellipticity (*e*) and liquid properties affect the residence time. The overall schemes were on the basis of the author’s earlier work [23] and several other studies for a prediction of drops colliding with a solid surface [27,28]. Water and two types of aqueous solutions (ethanol or glycerin) were employed as operating liquids, and air at room temperature and atmospheric pressure was employed as operating vapor. The volume fractions were represented by *ψ*_1_ and *ψ*_2_ for the two phases. The Navier–Stokes equations for mass (1) and momentum (2) conservation were solved in the computational domain as:

(1) $$\frac{\partial }{\partial t}\left(\rho \right)+\nabla \cdot \left(\rho \vec{v}\right)=0$$

(2) $$\frac{\partial }{\partial t}\left(\rho \vec{v}\right)+\nabla \cdot \left(\rho \vec{v}\vec{v}\right)=-\nabla p+\nabla \cdot \left[\mu \left(\nabla \vec{v}+{\left(\nabla \vec{v}\right)}^{T}\right)\right]+\rho \vec{g}+2\sigma \rho \gamma \nabla \psi_{2}/\left(\rho_{1}+\rho_{2}\right)$$

where $\rho =\psi_{2}\rho_{2}+\left(1-\psi_{2}\right)\rho_{1}$, $\mu =\psi_{2}\mu_{2}+\left(1-\psi_{2}\right)\mu_{1}$, and $\gamma =-\left(\nabla \cdot \vec{n}\right)$, that was the curvature in the liquid–vapor interface, where $\vec{n}$ was the unit vector normal to the surface. The volume fraction was governed by the advection equation as $\partial \psi /\partial t+\vec{v}\cdot \nabla \psi =0$. The interfacial tension was calculated for shape evolution of the drop, as shown in the source term of Equation (2) [29]. The volume tracking of the surfaces was estimated by using the VOF algorithm [30]. The spatial derivatives were calculated by using convective models [31]. The time step and maximal internal iteration were chosen as 1 μs and 30 per time step, respectively. The computational domain had a rectangular shape with a mesh resolution of at least 50 cells per drop diameter. To converge the velocity and pressure, a criterion of the normalized residual was set to 10^−5^. To weaken the effect of shape oscillation on the post-impact behavior, the initial drop shape was patched near the ridge. To predict the bouncing behavior and residence time on the ridged surface, a static contact angle model was introduced in the current work. Table 1 shows various liquid properties and advancing contact angles reported in several studies. The static contact angle model of the current study adopted the advancing angles as a contact angle in the simulation because the studies reported very small contact angle hysteresis (<4°).

To validate the numerical model, the maximum spreading diameters (*D_m_*) of spherical drops obtained numerically were compared with those obtained from experimental studies regarding viscous drop impact on flat surfaces [18]. The previous study showed that experimental data agreed well with scaling relations of *D_m_*/*D* ~ *We*^1/4^ for *P* < 1 and *D_m_*/*D* ~ *Re*^1/5^ for *P* > 1 reported by Clanet et al. [13]. The least-square fitting lines of the experimental data yielded the numerical coefficient of 0.83, as follows [18]:

*D_m_*/*D* = 0.83*We*^1/4^
*for P* < 1, (3)

*D_m_*/*D* = 0.83*Re*^1/5^
*for P* > 1 (4)

**Figure 1:**
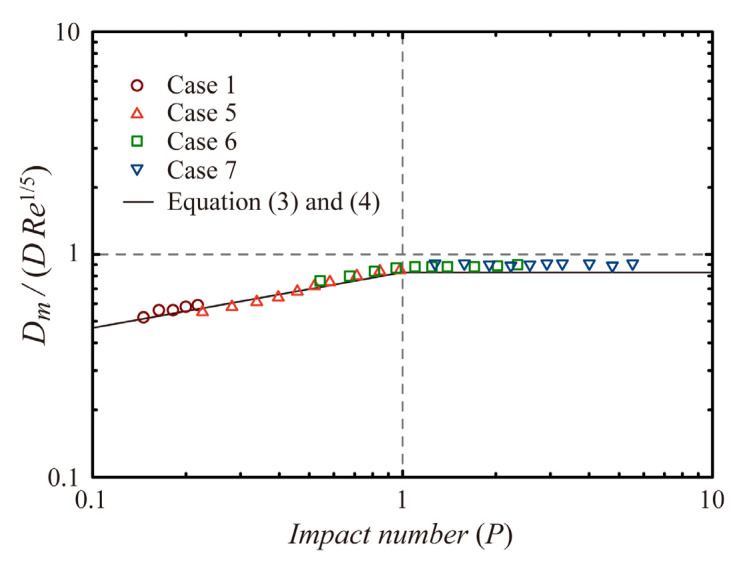
Validation of the numerical model based on the maximum spreading diameter (*D_m_*) of the spherical drop for viscous drop impact on non-wettable flat surfaces.

Figure 1 shows the numerical data of Case 1 (pure water drop) and Cases 5–7 (viscous drops) obtained from the current study and the fitting lines (Equations (3) and (4)) obtained from the previous experimental study. It was found that the numerical data agreed with the fitting lines of *D_m_* obtained experimentally. Moreover, the author’s previous study compared the residence times of ellipsoidal drops obtained numerically with those obtained experimentally to validate the numerical model for water drop impact on SHSs [25]. Numerical simulations have reasonably reproduced the residence times that depended on *e* and *We*.

**Figure 2:**
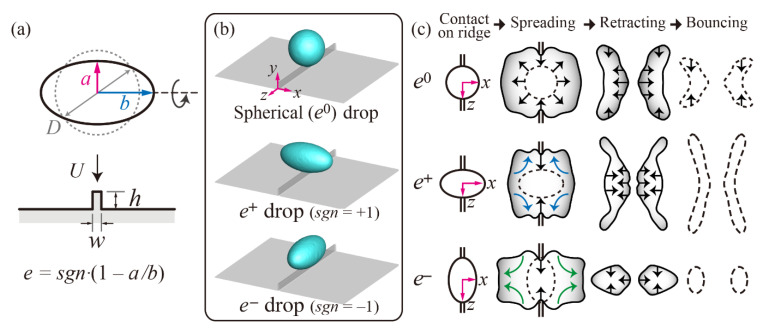
(**a**) Schematic of ellipsoidal drops impacting on non-wettable ridged surfaces with an initial velocity, *U*. The ellipsoidal drop’s ellipticity in the study is controlled as *e* = ±0.47. (**b**) Geometrical configurations of spherical and ellipsoidal (*e*^+^ and *e*^−^) drops with respect to the ridge. (**c**) Illustration of general evolution of water drop based on contact, spreading, retraction, and bouncing: reused from [25], Copyright (2021) with permission from Elsevier.

Numerical simulations confined the initial drop shape to prolate spheroids with half widths of *a* and *b* in the minor and major axes, respectively, as depicted in Figure 2a. The drop’s ellipticity was defined as *e* = *sgn*·(1 − *a*/*b*) for the following geometric relationship between the drop and ridge: *e*^+^ drops of which the major axis was orthogonal to the ridge line for *sgn* = +1 (namely, *e* > 0), and *e*^−^ drops of which the major axis was parallel to the ridge line for *sgn* = − 1 (namely, *e* < 0), respectively. The impact velocity used was *U* = 0.5–1.5 m/s. In the *x* direction, *x*_1_ and *x*_2_ were the half widths of the outer and inner rims, normalized by *D*/2, respectively. In the *z* direction, *z*_1_ was the half width of the film on the ridge, normalized by *D*/2. The drop’s ellipticity and normalized ridge’s width and height by *D* were controlled to *e* = ±0.47, *w* = 0.05, and *h* = 0.2 in the simulation, respectively.

## 3. Results and Discussion

Bouncing behavior of drops on the ridge surface can depend on the geometric configuration between the drops and ridge, as shown in the illustration of Figure 2b. After drops spread on the ridged surface, they are split into two parts by the ridge, which induces the formation of the inner rim to retract in the outward direction (away from the ridge). Finally, drops behave differently in the *x* and *z* directions during the retraction and then bounce off from the surfaces. *e*^+^ drops can offer an efficient way for decreasing the residence time (*t_c_*) noticeably compared with spherical and *e*^−^ drops. For example, *e*^+^ and *e*^−^ drops on the ridged surfaces decreased the residence time by approximately 55% and 38% below *e*^0^ drops on flat SHSs for pure water liquid at *We* = 47, respectively [25]. The findings were explained in terms of an initial mass distribution and a pronounced flow driven by the distribution during the spreading. The initial shape of the *e*^+^ drop intrinsically induces the pronounced flow outward in the *z* direction, which can evolve itself into widespread liquid along the ridgeline, as shown in the illustration of Figure 2c. In addition, after the split, the two parts start to retract outward and inward along the *x* direction, thereby leading to high aspect ratios of the liquids aligned on the *z* direction before the bouncing. In other words, a fast bouncing of the *e*^+^ drop can originate from a unidirectional retraction that induces the mass and momentum transfer from the *x* to the *y* directions, while the role of the *z* direction is negligible during the retraction. In contrast, the initial shape of the *e*^−^ drop intrinsically drives the pronounced flow outward in the *x* direction, which can evolve itself into widespread liquid along the direction perpendicular to the ridgeline, as shown in the illustration of Figure 2c. Thus, the *e*^−^ drops are considerably elongated in the *x* direction, and two fragments continue to move outward in the direction after the mother drop is split. For the asymmetric dynamics of the *e*^−^ drops, the roles of *x* and *z* directions in the mass and momentum transfer cannot be ignored. Accordingly, the shape evolution and residence time of the *e*^−^ drops show a striking contrast with those of *e*^+^ drops because the unidirectional retraction can be crucial for rapid bouncing.

Table 1 shows various liquid properties used in the current study, which correspond to ethanol or glycerin aqueous solutions in a certain weight percentage. Impact number (*P*) was used for a comprehensive estimation on whether capillary or viscosity governs the drop dynamics. In the current study, assuming that the impact velocity is constant, *P* increases exponentially with Case number (from Case 1 to Case 7). For example, the hydrodynamics of Case 1 (*P* = 0.02–0.1 and *Oh* = 0.002) and Case 4 (*P* = 0.1–0.4 and *Oh* = 0.008) can be determined by the inertia and capillary forces. For the highest viscosity, the hydrodynamics of Case 7 (*P* = 1.3–4.8 and *Oh* = 0.25) can be governed by the viscosity force.

**Figure 3:**
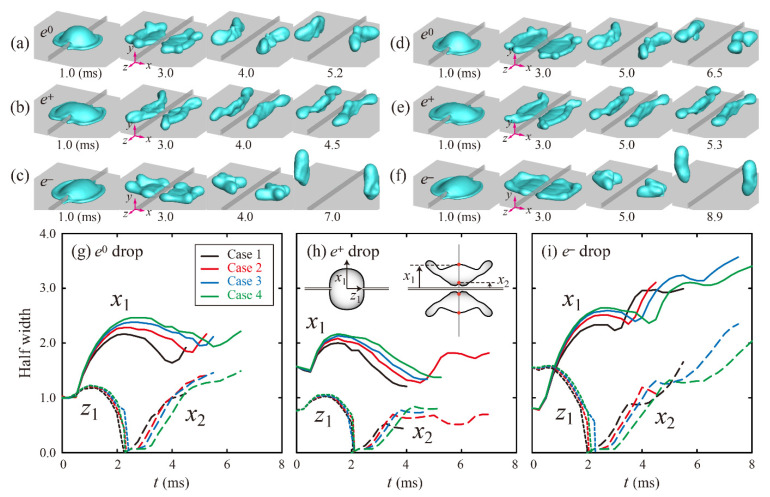
Snapshots of ethanol aqueous solutions of (**a**–**c**) 5 wt.% (Case 2) and (**d**–**f**) 20 wt.% (Case 4) at *U* = 1.3 m/s. The last snapshot for each *e* is captured at the moment of bouncing of drops from the surface and ridge. (**g**–**i**) Temporal variations in dimensionless half widths of *e*^0^, *e*^+^, and *e*^−^ drops for Cases 1–4. Solid, long dashed, and short dashed lines represent *x*_1_, *x*_2_, and *z*_1_, depicted in the inset of (**h**), respectively.

Shape evolutions of ellipsoidal drops with liquid properties and different drop sizes were investigated. First, snapshots of ethanol aqueous solutions (Cases 2 and 4) at a fixed impact velocity are shown in Figure 3a–f. Each last snapshot for *e*^0^, *e*^+^, and *e*^−^ drops was captured at the moment of bouncing of drops from the surface and ridge. The drops in Case 4 spread more widely than those in Case 2, as shown in the Figure 3a–f at 3 ms, because Case 4 has lower surface tension, lower contact angle, and higher viscosity than Case 2, according to the liquid properties. Solid, long dashed, and short dashed lines represent the temporal variation of the half widths, *x*_1_, *x*_2_, and *z*_1_, respectively, as depicted in the inset of Figure 3h. Temporal evolutions of the half widths for the four Cases indicate that the outer rims (*x*_1_) retract inward further to approach the inner rims (*x*_2_) as *P* decreases (from Case 4 to Case 1), as shown in Figure 3g–i. Evidently, low *P* can play an important role in shortening the residence time in a capillary-dominant regime.

**Figure 4:**
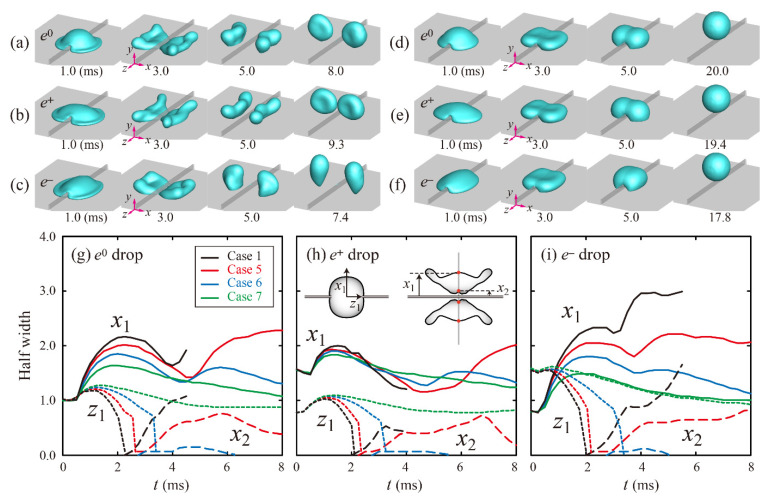
Snapshots of water/glycerin mixtures of (**a**–**c**) 60 wt.% (Case 5) and (**d**–**f**) 85 wt.% (Case 7) at *U* = 1.3 m/s. (**g**–**i**) Temporal variations in dimensionless half widths of *e*^0^, *e*^+^, and *e*^−^ drops for Case 1 and Cases 5–7.

High viscosities had a significant effect on altering the bounce dynamics and residence time. Figure 4 shows evolutions in shape and dimensionless half width for Cases 5 and 7 of water/glycerin mixtures, which reveal that a high *P* leads to small deformations in the spreading and retraction processes, compared with the drops in the capillary-dominant regime. Separated drops retract slowly and then bounce off near the ridge, as shown in Case 5 of Figure 4a–c. In addition, directly after drops are bouncing, contracted shapes along the *z* direction are found in *e*^0^ and *e*^+^ drops, whereas vertically elongated shapes are found in *e*^−^ drops. Meanwhile, drops are not split by a ridge and then evolve their shapes into spheroids directly after bouncing, as shown in Case 7 of Figure 4d–f. For high *Oh* (Cases 5–7), inertial and capillary forces only slightly affect the shape evolution, and viscous force is relatively dominant, which is different from the bouncing dynamics of drops observed at a low *Oh* (Cases 1–4).

**Figure 5:**
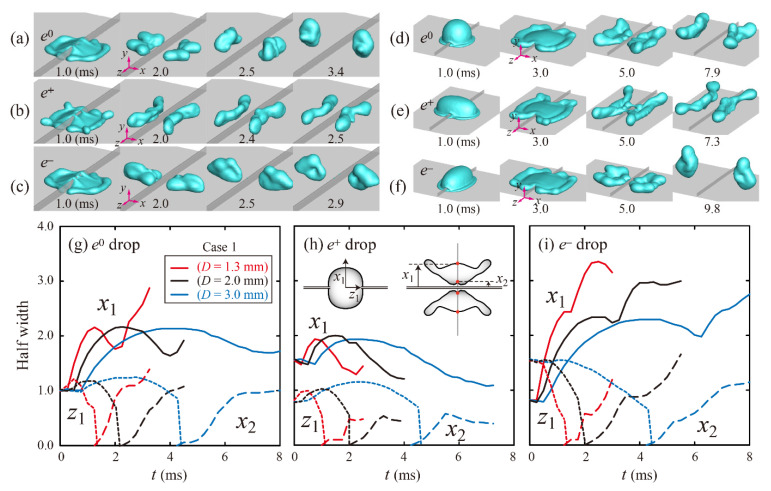
Snapshots of water drops of Case 1 with (**a**–**c**) *D* = 1.3 mm and (**d**–**f**) 3.0 mm at *We* = 47. (**g**–**i**) Temporal variations in dimensionless half widths of *e*^0^, *e*^+^, and *e*^−^ drops.

Snapshots of the drops with the diameters (*D*) of 1.3, 2.0, and 3.0 mm for Case 1 were obtained at the fixed *We*, as shown in Figure 5a–f. The shape evolutions of the two Cases exhibit distinct features of bouncing dynamics, such as a formation of liquid alignment on the *z* and *x* directions for *e*^+^ and *e*^−^ drops, respectively. This phenomenon leads to the significant reduction in *t_c_* of *e*^+^ drops because the newly formed inner and outer rims retract to the *x* direction, thereby inducing upward motions of the drops. Figure 5g–i indicate that the outer rims (*x*_1_) retract to the *x* direction toward the inner rims (*x*_2_) for *e*^+^ drops, whereas the outer and inner rims move further away from a ridge until drops are detached from the surface for *e*^−^ drops.

**Figure 6:**
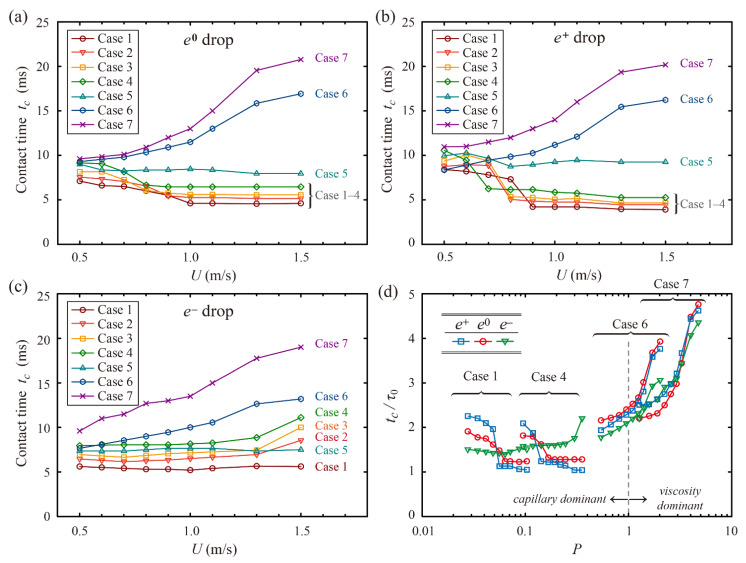
Effect of liquid properties on reduction in the residence time under various *U* for (**a**) spherical, (**b**) *e*^+^, and (**c**) *e*^−^ drops. (**d**) Residence time normalized by *τ*_0_ as a function of Impact number (*P* = *We*/*Re*^4/5^) for Cases 1, 4, and 6.

The residence time was predicted as a function of impact velocity for *e*^0^, *e*^+^, and *e*^−^ drops, as shown in Figure 6a–c, respectively. *e*^0^ and *e*^+^ drops show a constant decline in *t_c_* at low impact velocity, whereas *e*^−^ drops show no significant changes in *t_c_* at low impact velocity. In addition, *e*^0^ and *e*^+^ drops exhibit a substantial fall in *t_c_* for Cases 1–4 and no significant change at impact velocity above the thresholds, whereas *e*^−^ drops never decrease the residence time for any cases, although the impact velocity increases. It is found that *e*^0^, *e*^+^, and *e*^−^ drops on macro-ridge patterns cause 40–54%, 25–47%, and 0–35% reductions in residence time compared with *e*^0^ drops on flat surfaces at *U* = 1.3 m/s, respectively, which are obtained from Cases 1–4. For a moderate viscosity, the residence time of Case 5 only slightly changes with the impact velocity, whereas those of Cases 6 and 7 for high viscosities increase constantly, as shown in Figure 6a–c. The trend agrees with the fact that low *P* regime enables the mass redistributions of drops, which are important for reduced *t_c_*, such as the formation of a butterfly shape, whereas high *P* regime cannot form the shape properly. In other words, the viscous dissipation can retard the retraction process and suppress drop splitting, thereby leading the residence time to increase as the impact velocity increases.

**Figure 7:**
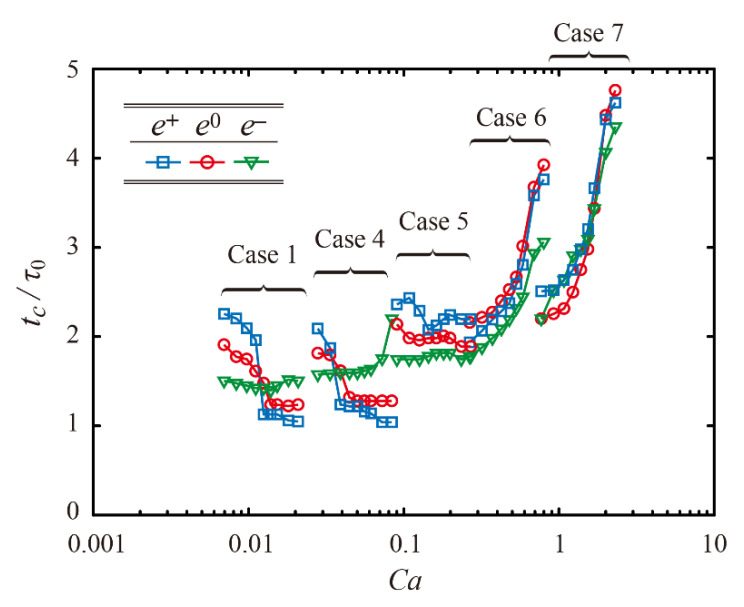
Normalized residence time as a function of *Ca*.

Figure 6d shows the residence time normalized by the initial-capillary time scale as a function of Impact number for Cases 1, 4, 6, and 7. Cases 1 and 4 exhibit almost similar changes in *t_c_*/*τ*_0_ with respect to *P*, which presents a striking contrast with Cases 6 and 7 showing a monotonic increase in *t_c_*/*τ*_0_. It is confirmed that Cases 1–4 under the capillary-dominant regime (*P* < 1) have a similar scenario of variation in *t_c_*/*τ*_0_. In the same manner, Cases 6 and 7 under the viscosity-dominant regime (*P* > 1) have a similar scenario of variation in *t_c_*/*τ*_0_. Hence, it is concluded that Impact number can govern whether a macro-ridge can lead to a steplike reduction in the residence time or not. Moreover, when the normalized residence time is plotted with the capillary number, it is found that there is a prerequisite of *Ca* < 0.4 for a fall decrease in the residence time to occur, as shown in Figure 7. The regime of small *Ca* indicates that the surface tension attains dominance over the viscosity.

**Figure 8:**
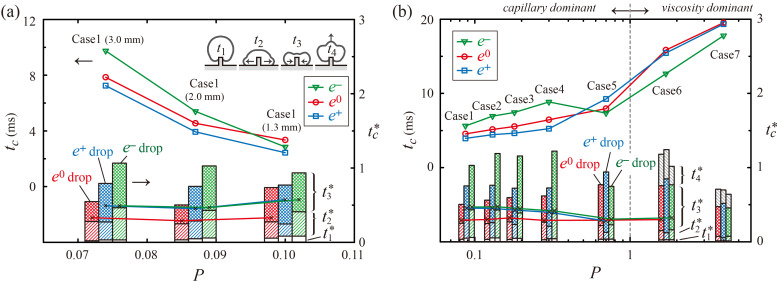
Effect of drop size and liquid properties on reduction in the residence time. (**a**,**b**) *t_c_* (symbol; left axis) and t_c_* (vertical stack; right axis) as a function of Impact number for (**a**) Case 1 with different *D* at *We* = 47 and (**b**) Cases 1–7 at a fixed impact velocity, *U* = 1.3 m/s. Solid lines within the stacks in (**a**,**b**) indicate the onset time of drop splitting.

To investigate the underlying mechanism behind the variation in residence time, t_c_* was defined as the residence time of ellipsoidal drops normalized by that obtained from the drop impact on a flat surface for the same *e*. In addition, t_c_* can be characterized in terms of several durations, *t*_1_*–*t*_4_*; that is, *t*_1_ is the duration for spreading along the ridge, *t*_2_ is the duration for spreading on the substrate, *t*_3_ is the duration for retraction on the substrate, and *t*_4_ is the duration for ascending the ridge, as depicted in the inset of Figure 8a.

To examine the effects of drop size and liquid properties on reduction in the residence time, *t_c_* (symbol) and t_c_* (vertical stack) were investigated as a function of *P*, as shown in Figure 8. The *t_c_* linearly decreases with an increase of *P* owing to a decrease in drop diameter, *D*, as shown in Figure 8a. The *e*^+^ drops exhibit the minimal *t_c_* among the drops in Case 1. The variation in t_c_* shows that a large *D* (low *P*) has a significant effect on the reduction in the residence time of *e*^0^ drops, whereas a small *D* (high *P*) has a significant effect on the reduction in the residence time of *e*^−^ drops, as shown in the cases of *D* = 3.0 and 1.3 mm of Figure 8a, respectively. The case of *D* = 1.3 mm presents a low deviation of t_c_* between *e*^0^, *e*^+^, and *e*^−^ drops.

Figure 8b indicates that *t_c_* slightly increases with Impact number at *P* < 1 (capillary-dominant regime), whereas *t_c_* greatly increases with Impact number at *P* > 1 (viscosity-dominant regime). In the capillary-dominant regime, *e*^+^ and *e*^−^ drops exhibit the minimal and maximal *t_c_* under Cases 1–4, respectively. Moreover, a low *P* has a substantial influence on the reduction in t_c_* of the *e*^0^ and *e*^−^ drops, as shown in Cases 1–4 of Figure 8b. Meanwhile, for Cases 5–7, the minimal *t_c_* appears at *e*^−^ drops. In the viscosity-dominant regime, *t_c_* increases significantly with *P* because *t*_4_, the duration for climbing up the ridge, mainly contributes to an increase in *t_c_*, as shown in Cases 6 and 7.

## 4. Conclusions

Numerical simulations were carried out for the bouncing behavior and residence time of elliptical footprint drops on the ridged surface using the VOF method. For low viscosities (Cases 1–4), *e*^0^ and *e*^+^ drops on macro-ridge patterns could cause at least 25% and 40% reductions in residence time compared with *e*^0^ drops on flat surfaces, respectively. For moderate viscosity, the residence time of Case 5 only slightly changed with impact velocity. For high viscosities (Cases 6–7), drops increased the residence time constantly so that incorporating a macroscopic texture would no longer promote a reduction in residence time. The trend agreed with the fact that the low *P* regime for impacts with *P* < 1 enabled the mass redistributions of drops, which were important for a fall reduction in residence time, such as the formation of the butterfly shape.

The asymmetric bouncing dynamics observed in the current work can be regarded as an initial value problem with liquid properties and drops’ ellipticity, compared with the conventional impact dynamics based on circular symmetry. This study provides insight on possible outcomes of impinging viscous drops on the ridged surfaces, and will help to develop the spraying system and superamphiphobic or SHSs for many applications, such as an anti-icing strategy during freezing rain or oil [10,19]. In addition, the ellipticity of drop shape is a controllable factor, which can help to adjust drop repellency from the textured surfaces in industrial applications, such as a dropwise condensation for enhancement of heat exchange performances [33].

Bouncing dynamics on non-wettable macro-textured surfaces can be extended to applications, such as environmental oil–water separation, waterproofing, and biomedical interactions. For example, the impact of core-shell or Janus drops on the surfaces can be used for a liquid separation method using the asymmetric bouncing dynamics induced by different liquid properties, such as density, viscosity, and interfacial tension [34]. In addition, bouncing dynamics on non-wettable macro-textured surfaces can also offer practical implications for designing water-repellent fibers or fabrics, such as hydrophobic fibers [35] and hair array [36,37].

## Figures and Tables

**Table 1:** Liquid properties used in the study.

Case	Test Liquids	*D* (mm) Equilibrium Diameter	*θ_a_* (°) Advancing Contact Angle	*ρ* (kg/m^3^) Density	*μ* (Pa s) Viscosity	*σ* (N/m) Surface Tension	Ohnesorge Number (*Oh*), (*μ*/(*ρDσ*)^1/2^)
1	Water [32]	2.0	170.3 ± 0.7	998.2	0.001	0.072	0.003
2	Water/ethanol (5 wt.%) [32]	2.0	167.1 ± 0.6	989.2	0.00125	0.0557	0.004
3	Water/ethanol (10 wt.%) [32]	2.0	168.4 ± 1.0	981.9	0.00152	0.0475	0.005
4	Water/ethanol (20 wt.%) [32]	2.0	164.5 ± 0.9	968.3	0.00212	0.0380	0.008
5	Water/glycerin (60 wt.%) [18]	2.0	163.4 ± 1.8	1150	0.0117	0.0648	0.029
6	Water/glycerin (75 wt.%) [18]	2.0	165.4 ± 0.8	1185	0.0339	0.0637	0.087
7	Water/glycerin (85 wt.%) [18]	2.0	163.2 ± 0.4	1210	0.0977	0.0635	0.249

## Data Availability

The data presented in this study are available on request from the corresponding author.
